# WiFi-Based Detection of Human Subtle Motion for Health Applications

**DOI:** 10.3390/bioengineering10020228

**Published:** 2023-02-08

**Authors:** Hui-Hsin Chen, Chi-Lun Lin, Chun-Hsiang Chang

**Affiliations:** 1 KLA Corporation, Chupei City 302, Taiwan; 2 Department of Mechanical Engineering, National Cheng Kung University, 1 University Rd., East Dist., Tainan 701, Taiwan; 3 Medical Device Innovation Center, National Cheng Kung University, Tainan 701, Taiwan

**Keywords:** channel state information, wireless sensor networks, health monitoring, motion detection, movement disorders

## Abstract

Neurodegenerative diseases such as Parkinson’s disease affect motor symptoms with abnormally increased or reduced movements. Symptoms such as tremor and hand movement disorders can be subtle and vary daily such that the actual condition of the disease may not fully present in clinical sessions. Health examination and monitoring, if available in the living space, can capture comprehensive and quantitative information about a patient’s motor symptoms, allowing physicians to make a precise diagnosis and devise a more personalized treatment. WiFi-based sensing is a potential solution for passively detecting human motion in a contactless way that collects no personally identifiable information. This study proposed an approach for human micromotion detection using the WiFi channel state information, which can be realized in a regular-sized room for home health monitoring and examination. Three types of motion were tested to evaluate the proposed method in quantifying micromotion using single and multiple WiFi links. The results show that micromotion could be captured at all distributed locations in the experimental environment (4.2 m × 7.9 m). Our computer algorithm computed the frequency and duration of simulated hand tremors with an average accuracy of 90.9% (single WiFi link)—95.7% (multiple WiFi links).

## 1. Introduction

In the last two decades, wireless technology has achieved significant success in data communication. Currently, WiFi connections exist in every houses due to their low cost and easy-to-set-up features, which also make wireless sensing using commodity WiFi devices a popular research field. In the past few years, human motion detection technologies using WiFi have been developed for indoor applications, such as localization/tracking [1], walking speed estimation [2], identity recognition [3], activity recognition [4], and fall detection [5].

Neurodegenerative diseases such as Parkinson’s disease (PD) are a progressive disease where patients’ motor symptoms develop slowly. As the global population ages, the prevalence of diseases is rapidly increasing. Unfortunately, there is no cure for the disease, and patients can only relieve symptoms and get back into the rhythm of life by taking drugs. Precise medication is critical to disease management but requires a comprehensive evaluation of the patient’s condition. However, assessing the motor functions of patients relies on the physician’s experience and subjective judgment; still, the disease’s actual condition may not be fully present during clinical examination due to the motor fluctuation or symptom variation in a day. Therefore, health monitoring is needed to detect motor symptoms and provide quantitative data.

At present, research on quantifying motor symptoms of PD mainly uses wearable devices and computer-vision-based methods. Sensing motion via wearable devices [6,7,8] has been well-established and is widely used owing to their high accuracy and low cost. However, wearing a device for long-term monitoring can be inconvenient or cause discomfort [9]. Patients should also be willing to wear such a device at any time. Although computer-vision-based systems [10,11] can be device-free, the patient’s privacy is compromised as recorded images need to contain facial and body features. Video filming is also restricted by its hardware capability and environmental settings [12]. WiFi-based sensing can be both device-free and private. Owing to the maturity of WiFi technology and its high-resolution signal transmission, WiFi channel state information (CSI) has shown high potential for developing wireless sensor networks [13,14,15]. Furthermore, the model-based WiFi CSI sensing methods establish a mathematical relationship between the target motion and signal fluctuations, which enhances data collection stability and environmental adaptability [16].

The main contribution of this paper is the realization and evaluation of the WiFi-based detection of micromotion related to PD symptoms. Abnormal gait, bradykinesia, and hand-resting tremor are typical symptoms of PD [17]. As studies have shown the capability of WiFi CSI in detecting walking [1,2,18], we target hand symptoms, including tremor and finger tapping, commonly observed/tested in the clinical examination. By taking advantage of WiFi sensing, which is contactless, not sensitive to lighting conditions, and easy to deploy, we aim to develop an assessment tool that can collect quantitative information on symptoms for physicians to make more accurate diagnoses.

## 2. Related Works

Micromotion detection for health monitoring is a popular research topic. Existing technologies are developed based on wearable- and smart-device-based computer vision algorithms and wireless signal-based methods.

### 2.1. Wearable and Smart Devices

Wearable devices such as accelerometers [7,19], gyroscopes [20], and force-sensitive resistors [6] are widely used owing to their low cost and high measurement accuracy. An increasing number of studies use commercial off-the-shelf products such as Wii^®^ Balance Board [8], iPhone [21], and smart watches [22] because of their accessibility. However, the device usually requires direct contact with the body for measurement, which can cause inconvenience, discomfort, and skin allergy [23], particularly in aged patients.

### 2.2. Computer-Vision-Based Methods

With the advancement of image processing techniques, quantifying motor symptoms based on computer vision has become increasingly popular [24,25,26]. Micromotion can be captured and quantified from the footage recorded to acquire relevant patient information [24,25]. However, the accuracy of these methods is extremely sensitive to lighting conditions [12]. Moreover, these methods typically require external objects (checkerboards [27], markers [28] on the body, etc. [29]) during video filming to achieve micromotion measurements. The privacy of the patient is another concern. Filming videos can capture personally identifiable information of a patient, such as facial and body features. In addition, professional assistance would be required to set up a computer-vision-based method at home.

### 2.3. Wireless-Signal-Based Methods

Non-contact wireless sensor networks include ultrasound [30], infrared (IR) [31], and radio frequency (RF) [32]. RF methods can be subdivided into frequency modulation (FM) [33], ultra-wide band (UWB) [34], WiFi received signal strength indication (RSSI) [35], and WiFi CSI [36]. The two WiFi-based methods (i.e., via RSSI and CSI) have low deployment costs, as their infrastructure exists in almost every home today. More importantly, no device needs to be worn by the target person, and no personally identifiable information is acquired. Compared to RSSI, CSI can provide better granular information, leading to a higher resolution of captured data.

The CSI tool for 802.11n NIC Intel 5300 was released in 2010 [37]. From that time, CSI has been widely used for environmental detection and human activity recognition, including large-scale movement (e.g., daily activities [38], falling [5], localization [39], and human identification [40]) and subtle scale motion (e.g., gestures [41], shape of mouth [42], chewing and swallowing [43], and breathing [44]).

Establishing a relationship between movements and CSI is the key to motion quantification. In the early development of CSI-related research, pattern-based sensing methods [1] were mostly used. For activity recognition, the pattern-based method collects CSI data, extracts distinctive features of a target motion, and classifies activity types via manual analysis [5], machine learning, or deep learning methods [45]. However, these types of methods lack adaptability to different environments, which means that the same algorithm may not be applicable with changing environmental configurations. Consequently, researchers have begun to develop model-based sensing methods that aim to interpret the mathematical relationship between motion and signal fluctuations using a physical model; for example, the Fresnel zone [46], angle of arrival (AOA) [47], CSI speed model [48], and CSI activity model [48]. To date, very few studies have investigated the quantification of motor symptoms in Parkinson’s disease using WiFi CSI-based methods. Some pattern-based sensing methods have been developed to study the freezing of gait [49] and pill-rolling tremors [50]. However, no model-based method has thus far been found to quantify any of these motor symptoms.

This paper proposes a WiFi-based method that utilizes the CSI and Fresnel zone theory to quantify the most typical hand motor symptoms in PD, including resting tremor and finger tapping, in Parkinson’s disease. The algorithm processes raw CSI data and eliminates environmental interference to the signal, allowing the subtle motion to be detected throughout the experimental environment. We also designed experiments in a room with WiFi signal coverage to demonstrate the symmetric properties of single and multiple Fresnel zones, which were used to derive the contour of the detection accuracy. Finally, both settings of single and multiple WiFi links were evaluated to further our understanding of the WiFi CSI sensing ability. The strategy for using multiple WiFi links in a larger room was also discussed, moving the technology closer to a realistic application.

## 3. Materials and Methods

### 3.1. Overview

In this study, the physical layer CSI was utilized as a primary indicator for human motion. When a WiFi signal propagates along multiple paths in an environment, a moving target would affect a fraction of propagation paths and be depicted by CSI variations. We considered symptoms related to hand movement as target motion and hypothesized that such movement would cause temporal variations in CSI, which can be collected from commodity WiFi devices.

To describe the basic model of CSI [48], the wireless channel in the frequency domain should be defined first:

(1) Y=H×X+N

where H is the channel matrix of CSI; Y and X are the received and transmitted signal vectors, respectively; N is an additive white Gaussian noise vector. Therefore,

(2) $$H\left(i\right)={\left|H(i)\right|e}^{jsin∠H(i)}$$

where H(i) represents the value of CSI for the *i*-th subcarrier and $\left|H(i)\right|$ and ∠H(i) are the amplitude and phase of the *i*-th subcarrier.

The proposed method detects the variations in CSI amplitude, phase, and phase difference to quantify the target motion. The method was evaluated by four scenarios for the basic signal selection, directional effect of motion, and sensing accuracy.

### 3.2. Data Processing

**Figure 1:**
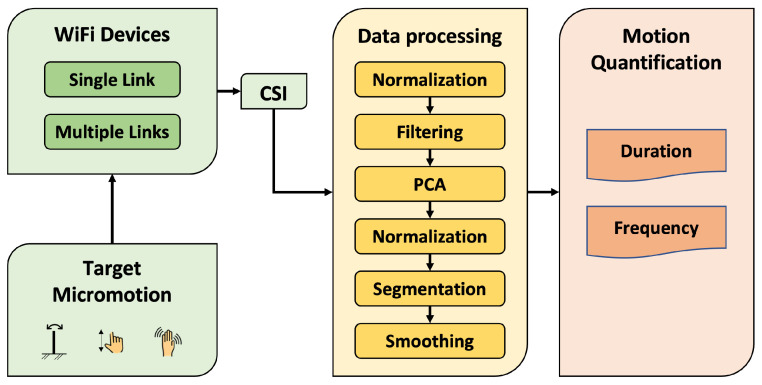
The proposed CSI data processing procedures.

We set up WiFi devices to collect CSI data when target motion was performed. The experimental settings and WiFi device configurations are explained in Section 3.4.1 and Section 3.4.2, respectively. The collected CSI data were processed through several procedures, as shown in Figure 1.

Firstly, given an input raw CSI sequence *P* of amplitude, phase, or phase difference, we performed Z-score normalization to obtain $P^{\prime }$, as shown in Equation (3), where $\bar{P}$ and σ are the average and standard deviation of *P*, respectively.

(3) $$P^{\prime }=\frac{P-\bar{P}}{\sigma }$$

We then used a third-order Butterworth filter [51] to remove undesired noise, which could be due to the environment or hardware, from the normalized CSI sequence. A lower cutoff frequency, $f_{l}$, of 3 Hz and a higher cutoff frequency, $f_{h}$, of 6 Hz [52] were used for processing the resting tremor test data. The test data of steel ruler vibration and finger tapping were processed by cutoff frequencies of [1, 10] Hz. The transfer function of the third-order Butterworth bandpass filter is shown in Equation (4), where coefficients $a_{i}$ and $b_{i}$ are determined by selected filtering parameters, including polynomial order and cutoff frequencies. After filtering, the output CSI sequence *A* can be obtained (Equation (5)).

(4) $$G\left(z\right)=\frac{\sum_{i=0}^{2n}b_{i}\;\cdot \;z^{-i}}{\sum_{i=0}^{2n}a_{i}\;\cdot \;z^{-i}}$$

(5) $$A\left(z\right)=P^{\prime }\left(z\right)\;\cdot \;G\left(z\right)$$

Secondly, we performed principal component analysis (PCA) [48] to find the best presentation of signal response to the target motion. The reason for this step was that the CSI contained data in 180 subcarriers (this is explained in Section 3.4.1), but only some of them provided useful information related to the target motion. Singular value decomposition (Equation (6)) was used to decompose *A* into the principal components *V*, and the best principal component $V_{*}$ was determined as the one with the largest variation in the normalized amplitude of the data. Another normalization was applied to $V_{*}$ to yield $V_{*}^{\prime }$

(6) $$A=U\Sigma V^{T}$$

**Figure 2:**
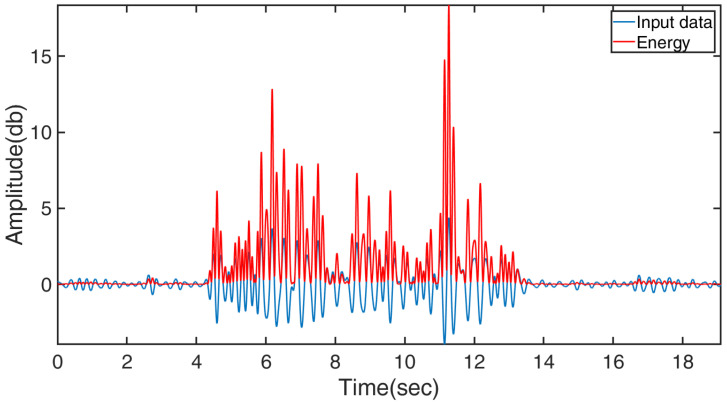
A sample of CSI sequence and calculated energy.

Thirdly, the short-time energy threshold segmentation [53] was used to distinguish between the active and rest states, as shown in Equation (7), where *E* represents energy of the input data series (i.e., $V_{*}^{\prime }$), w(n−m) is the window, *n* is the sample that the analysis window is centered on, and N is the window length (set as 51 in this study). A sample result of energy calculation is illustrated in Figure 2, showing that the active state can be easily identified from the energy plot by a basic thresholding technique to obtain the data segment of the target motion ($V_{W}$).

(7) $$E_{n}=\sum_{m=n-N+1}^{n}{\left[V_{*}^{\prime }\left(m\right)w\left(n-m\right)\right]}^{2}$$

Finally, a third-order Savitzky–Golay filter [54] with a window length of 101 was used to smooth the data and remove fake peaks. The parameters of the Savitzky–Golay filter were determined by experiments. We first confirmed that the third-order filter would properly smooth out the data while best preserving true peaks. Then, four different window lengths, 51, 101, 151, and 201, were evaluated. Within the range of these values, the results showed that both window lengths 51 and 101 achieved the best accuracy. The accuracy decreased when the window length increased from 101 to 201, and the decrease was 8% when the window length reached 201. The equation of the third-order Savitzky–Golay filtering is shown in Equation (8), where *j* is the index number of the input and output data sequences ($V_{W}$ and *Q*), and the convolution coefficients, $C_{i}$, are determined by a given set of polynomial order and window length *m*.

(8) $$Q\left(j\right)=\sum_{i=\frac{1-m}{2}}^{\frac{1+m}{2}}C_{i}V_{W_{j+i}}$$

An input CSI sequence contained 20-s data in 180 subcarriers with a sampling rate of 1000 Hz (the details are explained in Section 3.4.1). The size of the output sequence in the data processing remained the same until the segmentation (see Figure 1). The size of the output sequence in the segmentation step, $V_{W}$, depended on the start and end locations identified for the target motion. The $V_{W}$ was then smoothed, and the output sequence length was the same as $V_{W}$. Table 1 shows a summary of all parameters used in the CSI data processing.

### 3.3. Motion Quantification

The processed data would contain enhanced signal responses to the motion such that the wave pattern induced by the hand movements or ruler vibration could be easily identified. Thus, the movement quantity of motion, i.e., numbers of vibrations, finger taps, and tremors, in a piece of recorded data could be computed from the number of wave crests. The duration and frequency could also be calculated.

It is crucial to correctly identify peaks in the CSI sequence corresponding to the motion. To ensure consistent and accurate quantification results, our method used three main criteria for finding peaks: minimum separation between peaks, minimum peak height, and minimum peak prominence. The minimum separation is the most important criterion for excluding fake peaks that occur in too small intervals, which can be determined according to the frequency range of the target motion and WiFi sampling rate. For example, the upper frequencies of finger tapping and resting tremor were 10 and 6 Hz in our tests (Table 1), and the sampling rate was 1000 Hz; therefore, the minimum separation was calculated to be 100 data points for finger tapping and 166 data points for resting tremor.

Peaks that satisfy the minimum separation could still be fake ones, which occasionally appear in the static status due to environmental noise. The minimum peak height and peak prominence were used to avoid finding those fake peaks. The former eliminates extremely low peaks; the latter measures how much a peak stands out due to its intrinsic height and location relative to other peaks. By observing those peaks caused by the environmental noise in the collected data, we set the minimum peak height as 0.2 max(Q) and the minimum peak prominence as 0.3 max(Q). These empirically determined parameters helped to improve the peak finding accuracy by 3–5% compared to the cases that only used the minimum separation. With all criteria, although there were still true/fake peaks identified incorrectly, the error rate of motion quantification was within 7% compared to the reference data (explained in Section 3.4.2) for the most challenging case (resting tremor).

### 3.4. Experiments and Evaluation

#### 3.4.1. Experimental Setup

**Figure 3:**
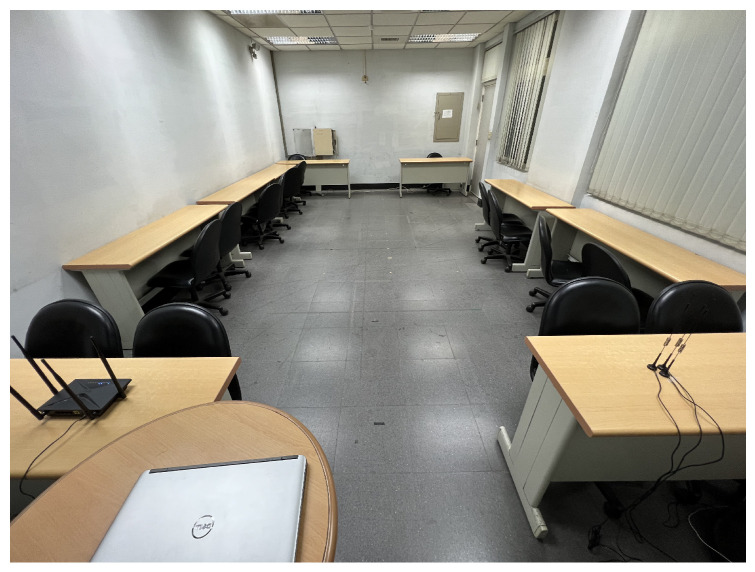
The university discussion room where the experiments were conducted. The photo shows the experimental setting of a single WiFi link. The Tx and Rx are placed on separate desks (0.75 m high).

Experiments were conducted using the Linux 802.11n CSI Tool [37] with commodity devices, including a TP-link Archer C60 wireless router as transmitter (Tx) and a Dell E6440 laptop as receiver (Rx). The network interface controller operated at a 5 GHz frequency band with a transmission rate of 1000 Hz. There were three antennas on Rx and two antennas on Tx to obtain up to six CSI streams, and each stream contained 30 subcarriers. They all record the environmental information. The experimental environment was an enclosed university discussion room (4.2 m × 7.9 m) with existing desks and chairs (Figure 3).

#### 3.4.2. Evaluation and Verification

**Figure 4:**
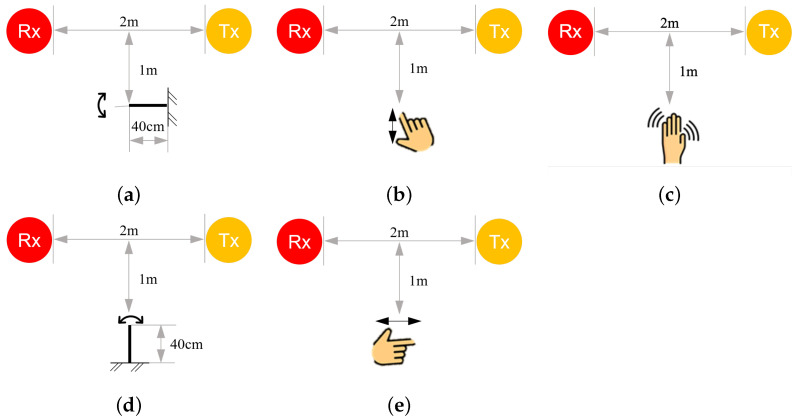
Experimental deployment of the three types of motion, including steel ruler vibration, finger tapping, and hand resting tremor. (**a**) The steel ruler vibration perpendicular to LoS. (**b**) The finger tapping perpendicular to LoS. (**c**) Hand resting tremor. (**d**) The steel ruler vibration parallel to LoS. (**e**) The finger tapping parallel to LoS.

The proposed method was evaluated using three types of motion: steel ruler vibration, finger tapping, and resting tremor (Figure 4). The steel ruler vibration (Figure 4a) was used to evaluate the sensing capability as gradual attenuation of motion is produced by the damping oscillation of a cantilever beam. The ruler was given a 10 cm initial displacement at the free end and released to vibrate until it returned to the static condition. The finger tapping (Figure 4b) required a person, following the metronome sound, to tap the tip of the index finger against the tip of the thumb using the right hand at a frequency of approximately 1 Hz. The measured range of finger displacements was approximately 8 cm. To mimic the resting tremor (Figure 4c), a person’s palm moves back and forth about the axis of the arm by rotating the wrist with random frequencies in the range of 3–6 Hz (similar to the resting tremor of PD [52]) and a hand fluctuation of approximately 8–10 cm radially. The total duration of each test was approximately 20 s. For the steel ruler vibration, the ruler was stationary for the first 5 s and then was released to start the motion in the 6th second. For finger tapping and resting tremor, the person’s hand stayed stationary for the first 5 s and started to move from the 6th second for 10 s. By default, a single WiFi link was used, and the target motion took place 1 m away from the midpoint of the line of sight (LoS) and as high as the Tx-Rx height (0.75 m).

Understanding the characteristics of target motion would help to capture correct wave peaks in the signal and avoid false ones. The frequency and duration of the three types of motion were described in Table 2, as these two parameters were of interest in this study. The information was used to confirm that the signal of motion was received correctly; more specifically, it was used to determine the minimum separation for identifying fake peaks.

**Figure 5:**
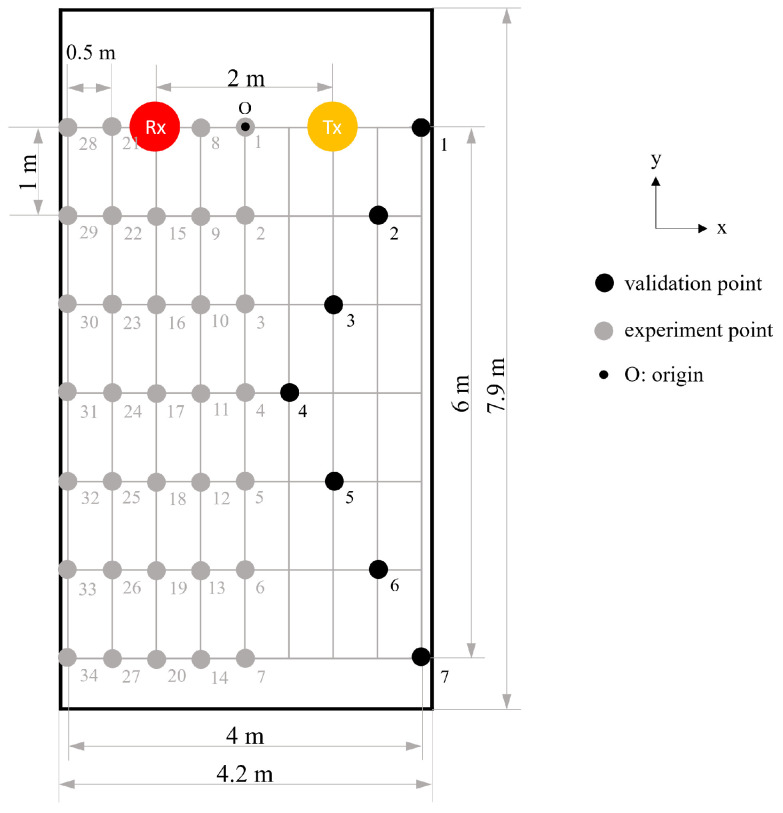
Experimental deployment for detecting the resting tremor using a single WiFi link.

**Figure 6:**
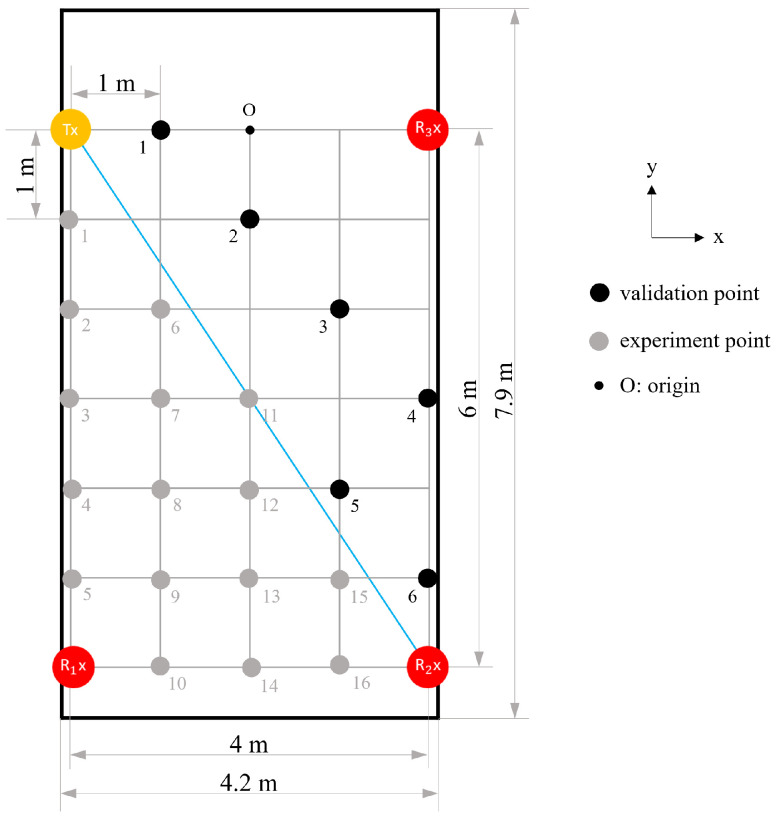
Experimental deployment for detecting the resting tremor using a transmitter paired with multiple receivers.

The proposed method was evaluated in the following four scenarios.

a. **Determine the basic signal for motion analysis:** For the experimental deployment shown in Figure 4a–c, we examined the responses of basic signals, such as amplitude, phase, and phase difference, to determine which best captured the target motion. This scenario used the default settings, i.e., the target motion took place 1 m away from the midpoint of the LoS and was detected by a single WiFi link.
b. **Understand the directional effect of motion:** We changed the direction of motion to be parallel to the LoS of the WiFi link (in such a case, the CSI variation would be minimal according to the Fresnel zone theory) and evaluated the sensing performance (Figure 4d,e). No change in the direction of motion was necessary for the resting tremor test as it involved motion in all directions. This scenario also used the default settings.
c. **Evaluate the sensing accuracy of a single WiFi link:** With a single WiFi link arranged as in Figure 5, a healthy person, the only one in the room, imitated the hand resting tremor. By defining the midpoint of the LoS as the origin, the target motion was performed at 34 experimental points (gray dots) spread over the left-hand side of the room. These points were arranged on a square grid with a 1 m interval. The impact of X and Y distances between the location of motion and the origin on the sensing accuracy was studied. The results were used to establish a sensing accuracy model on the left-hand side of the room, which could be mapped to the other side of the room based on the symmetric property of the Fresnel zone (about the centerline of the LoS). To verify that, additional tests were performed at seven validation points (black dots) on the right-hand side of the room, and the results were compared with those obtained by the symmetric mapping. The test was repeated eight times at each experimental/validation point.
d. **Evaluate the sensing accuracy of multiple WiFi links:** Similar to the scenario that evaluated a single WiFi link, the arrangement of WiFi devices changed to one Rx and three Txs at the four corners of the room (Figure 6). With the origin defined at the same position in the room, the target motion was performed at 16 experimental points (gray dots) spread over the lower-left triangle area of the room. Again, based on the symmetric property of the Fresnel zone, we created a sensing accuracy contour over the entire room by mapping the accuracy model of the lower-left triangle area to the upper-right triangle area. For verification purposes, additional tests were performed at six validation points (black dots) in the upper-right triangle area, and the results were compared with those obtained by the symmetric mapping. The test was performed eight times at each experimental/validation point.

The detailed experimental settings and types of motion used in each scenario are summarized in Table 3.

**Figure 7:**
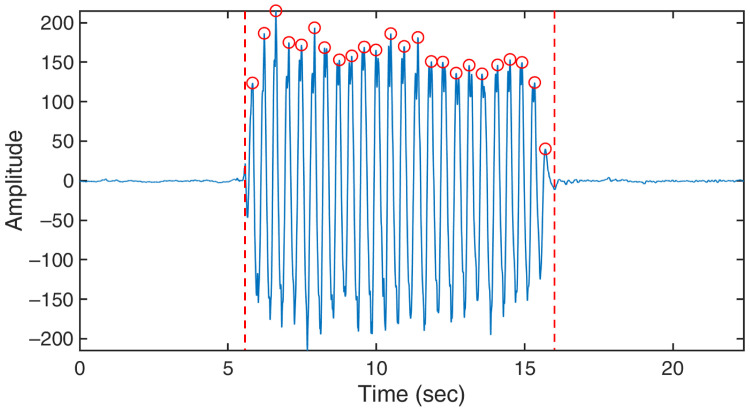
A sample reference data collected from a resting tremor test. The red circles indicate peaks in the data identified by our algorithm and are used to count the number of movements.

In all tests, a wearable sensor with a six-axis accelerometer and gyroscope (MetaMotionC, MBIENTLAB Inc., San Francisco, CA, USA) was simultaneously used to capture reference data at a sampling rate of 200 Hz. The sensor was attached to the free end of the steel ruler to detect the vibration. For resting tremor, the person who performed the task wore the sensor on the wrist. The sensor was not used in the finger-tapping test as the person already followed the metronome sound at 1 Hz. The reference data served as the basis of comparison for evaluating the accuracy of our WiFi-based sensing in terms of the frequency and duration of motion. A sample reference data collected from a resting tremor test is shown in Figure 7.

The absolute error was defined as the discrepancy between the frequency/duration of motion quantified using the CSI data, *q*, and that quantified using the sensor measurements, *g*. Therefore, the error percentage could be calculated by:

(9) $$e=\frac{1}{N}\sum_{i=1}^{N}\frac{\left|q_{i}-g_{i}\right|}{g_{i}}\times 100\%,$$

where *N* is the number of repetitions for a test item. The accuracy would be 1−e.

## 4. Results

### 4.1. Scenario a: Best Basic Signal for Motion Analysis

**Figure 8:**
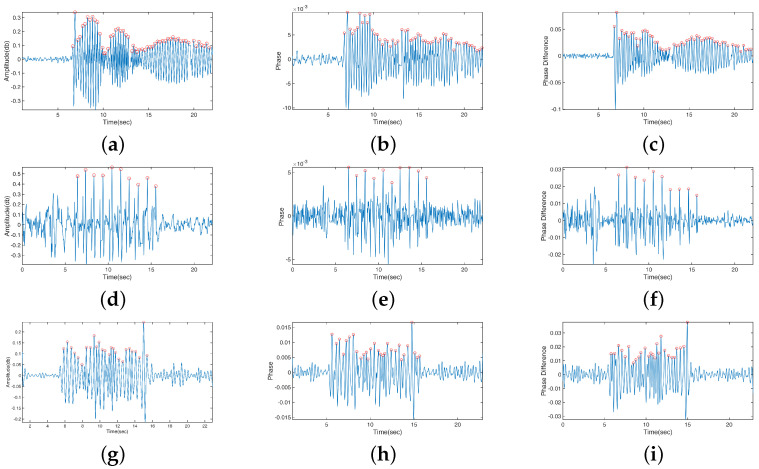
The processed CSI data of target motion from the three types of basic signals. The red circles indicate peaks in the data identified by our algorithm and are used to count the number of movements. (**a**) Steel ruler vibration—amplitude. (**b**) Steel ruler vibration—phase. (**c**) Steel ruler vibration—phase difference. (**d**) Finger tapping—amplitude. (**e**) Finger tapping—phase. (**f**) Finger tapping—phase difference. (**g**) Resting tremor—amplitude. (**h**) Resting tremor—phase. (**i**) Resting tremor—phase difference.

All basic signals, including amplitude, phase, and phase difference, clearly captured the effects of steel ruler vibration, finger tapping, and resting tremor (Figure 8). These results were further compared with the reference data. In the steel ruler vibration, the CSI amplitude (Figure 8a) response calculated the number of vibrations with an accuracy of 96.2%, whereas the CSI phase (Figure 8b) and phase difference (Figure 8c) estimated with 100% accuracy. Regarding the finger tapping, all three basic signals perfectly captured the same number of taps as those detected in the reference data. The amplitude (Figure 8d), phase (Figure 8e), and phase difference (Figure 8f) data estimated the duration of motion with 98.7%, 95.6%, and 95.3% accuracy, respectively. In resting tremors, the accuracy of detecting the number of movements was 91.7% using either amplitude (Figure 8g) or phase difference data (Figure 8i), and 79.2% using phase data (Figure 8h). Furthermore, the accuracy of duration estimation was 97.3%, 99.9%, and 91.7% using amplitude, phase, and phase difference, respectively.

The amplitude is a directly extracted measure of CSI, which is the most used basic signal. The CSI amplitude represents the decay of the signal strength caused by the multipath effect. The CSI phase also records changes in the wireless signal to reflect activities that occur in the environment. However, issues such as the carrier frequency offset and sampling carrier frequency offset occurred because of force majeure [55]. The CSI phase difference contains information between adjacent antennas. Both phase and phase differences must be sanitized before signal processing. Our results showed that the amplitude, phase, and phase difference could effectively sense steel ruler vibration, finger tapping, and resting tremor with satisfactory accuracy in motion quantification. Therefore, we used the CSI amplitude for motion quantification in the remaining experiments to optimize the computational efficiency and sensing accuracy.

### 4.2. Scenario b: Directional Effect of Motion

**Figure 9:**
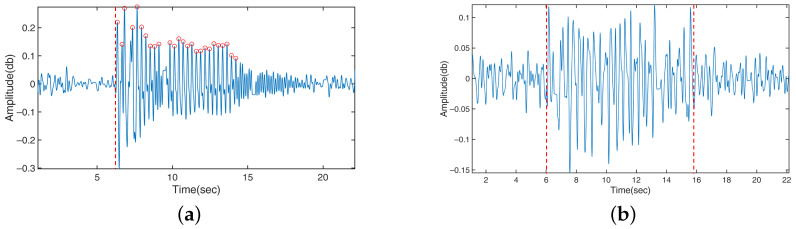
The processed CSI data of the target motion parallel to LoS demonstrates the directional effect. The red circles in (**a**) indicate peaks in the data identified by our algorithm and are used to count the number of movements. Peaks are not annotated in (**b**) as they are poorly found by the algorithm due to the directional effect of motion. (**a**) Steel ruler vibration. (**b**) Finger tapping.

Using the variation in the CSI amplitude, the directional effect of motion was investigated by comparing the sensing performance in the five experimental settings shown in Figure 4. For steel ruler vibration, the accuracy of frequency estimation was 96.2% when the motion was perpendicular to the LoS (Figure 8a) and was 86.4% for the parallel case (Figure 9a). As for finger tapping, the number of taps could only be counted correctly when the direction of motion was perpendicular to the LoS, with an accuracy of duration estimation of 98.7% (Figure 8d). When the direction of motion was parallel to the LoS, the CSI variation became much smaller (Figure 9b), making it difficult to quantify the number of taps. Our algorithm could only identify a rough time region with relatively large magnitudes of CSI, which indicated the duration of motion (the accuracy of duration estimation was 97.6%). The resting tremor involved hand movements in all directions. Consequently, the motion was well quantified (Figure 8g), regardless of the arm orientation. The accuracy of frequency and duration estimation was 91.7% and 97.3%, respectively. Table 4 summarizes the sensing performance of the three types of motion perpendicular and parallel to the LoS. The duration of steel ruler vibration was not detected, as the motion would gradually decay to very small magnitudes that exceed the detectable range of WiFi CSI. The main purpose of the steel ruler vibration test was to understand the ability of the proposed approach in detecting small motion with known magnitudes. The sensing performance was comprehensively evaluated for the two types of hand movements: finger tapping and hand resting tremor. However, the frequency of finger tapping parallel to LoS could not be quantified due to the directional effect.

### 4.3. Scenario c: Sensing Accuracy of a Single WiFi Link

Among the 34 experimental points in the test environment (Figure 5), the average sensing accuracy of the motion frequency was 90.2%, with a standard deviation of 3.6%. The first row of experimental points (1, 8, 21, and 28) obtained the best performance, with the highest accuracy of 96.2% at Point 1. The last row of experimental points (7, 14, 20, 27, 34) exhibited the worst performance, with an accuracy of 84.0% at Point 34.

**Figure 10:**
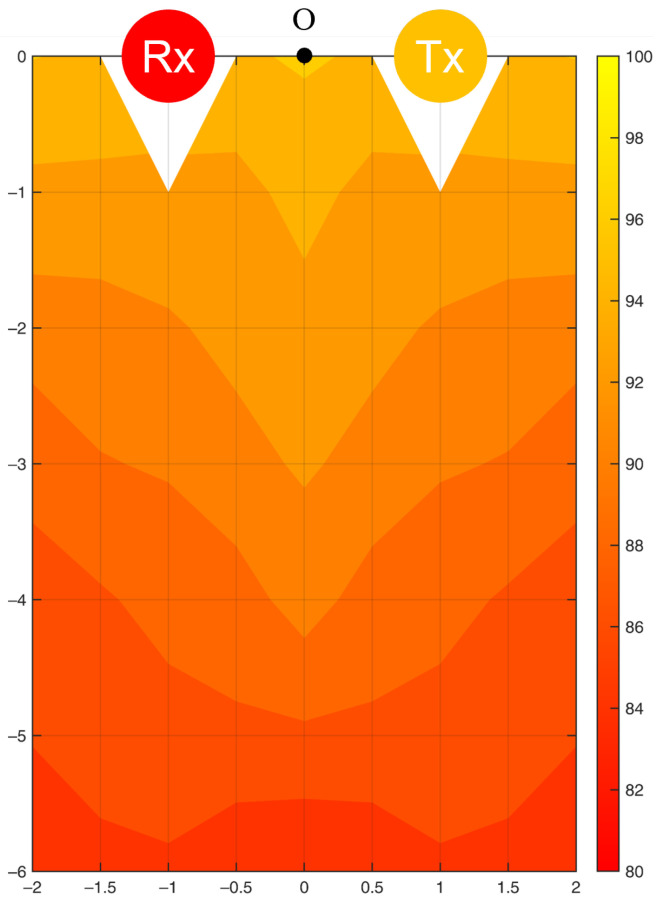
Accuracy contour (%) in detecting the frequency of resting tremor by a single WiFi link.

A filled contour of the sensing accuracy for the frequency of motion is shown in Figure 10. The left half of the contour was plotted by interpolating the results of 34 experimental points (Figure 5) and is mapped to the right half under the assumption of the symmetric property of the Fresnel zone. A single WiFi link formed only one Fresnel zone in space such that the accuracy contour would be symmetric to the centerline of the LoS. To verify the assumption, we evaluated the sensing accuracy at the seven validation points and compared it with the symmetric model. The errors were within 1.5%, which proved the symmetric property of the Fresnel zone for a single WiFi link.

**Figure 11:**
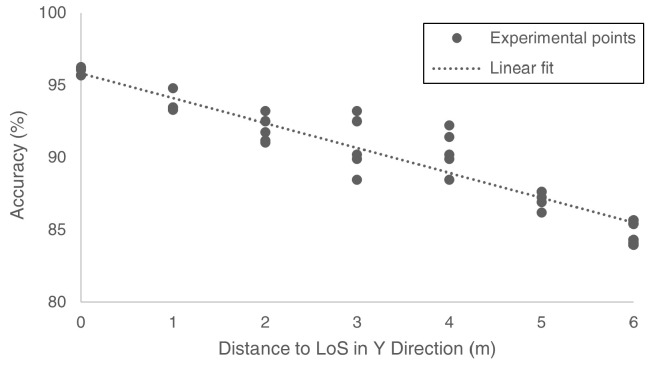
The sensing accuracy for the frequency of motion yielded at the 34 experimental points versus their distance to origin in the Y direction and the calculated linear relationship.

The effect of distance between the target motion and the origin on the sensing accuracy was much more clearly observed in the Y direction than in the X direction. Figure 11 shows the correlation between the sensing accuracy for the frequency of motion (*a*) yielded at the 34 experimental points and their distance to the origin in the Y direction ($d_{y}$). We calculated a linear relationship (Equation (10)) using the linear least-squares method [56], which suggested that *a* decreases as $d_{y}$ increases by a coefficient of −1.72%/m.

(10) $$a=-1.72d_{y}+95.82;R^{2}=0.89$$

**Figure 12:**
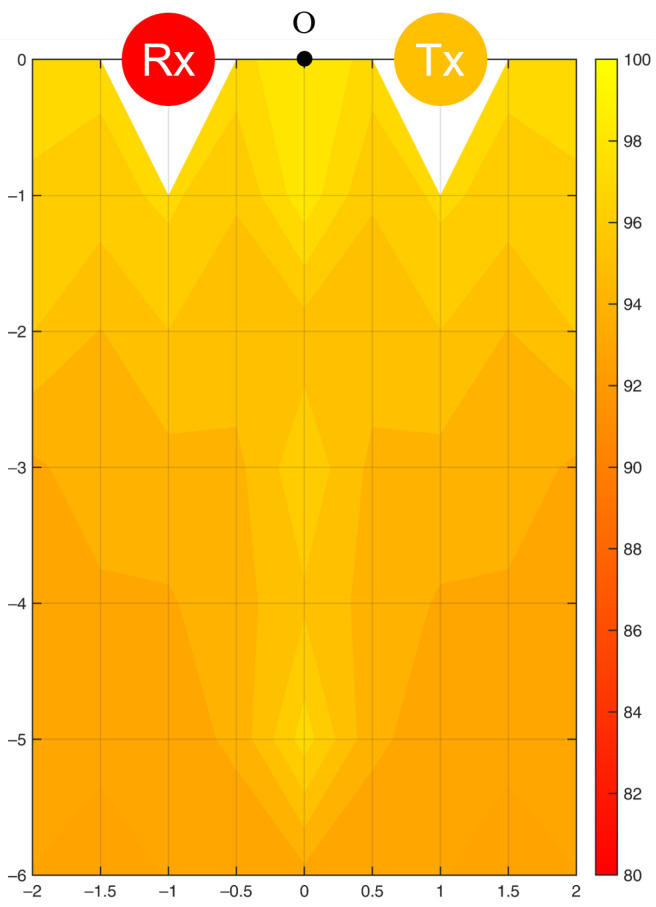
Accuracy contour (%) in detecting the duration of resting tremor by a single WiFi link.

For the duration of motion, the highest sensing accuracy was 99.0% (Point 28), and the lowest was 92.7% (Point 14), with an average of 95.2% and standard deviation of 1.75%. Figure 12 illustrates the filled contour. Although the sensing accuracy dropped as the detection point moved away from the LoS, the distance effect was less significant in both the X and Y directions. In the test environment, the duration of motion was better identified than the frequency.

### 4.4. Scenario d: Sensing Accuracy of Multiple WiFi Links

**Figure 13:**
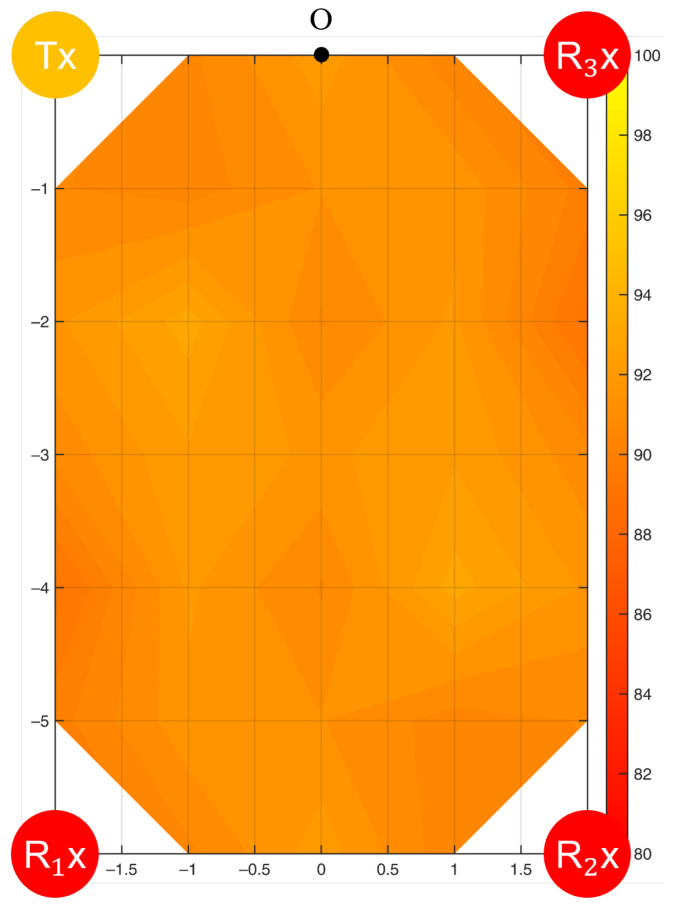
Accuracy contour (%) in detecting the frequency of resting tremor by multiple WiFi links.

In this setup, a Tx and three Rxs at the four corners of the room formed three WiFi links. Compared to the single WiFi link, the average sensing accuracy for the frequency of motion increased from 90.2% to 91.4% (standard deviation: 0.93%). The highest accuracy was 93.2% at Point 6, and the lowest was 89.3% at Point 4. Figure 13 illustrates the filled accuracy contours.

**Figure 14:**
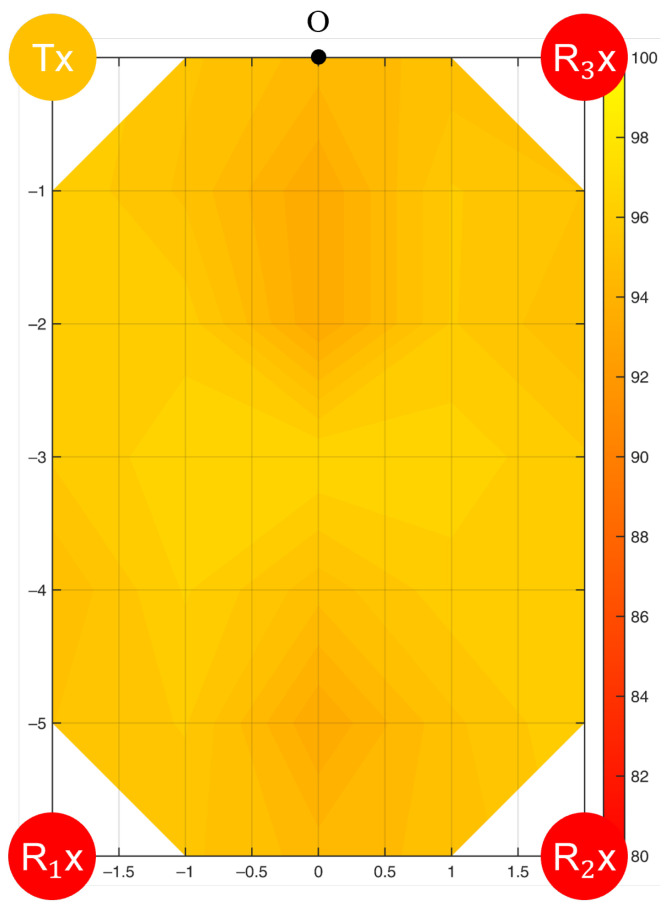
Accuracy contour (%) in detecting the duration of resting tremor by multiple WiFi links.

For the duration of motion, the highest sensing accuracy was 97.0% at Point 11, the lowest was 93.5% at Point 13, and the average was 95.7%, with a standard deviation of 0.86%. The filled accuracy contours are shown in Figure 14. Additionally, the sensing accuracy improved by 1.8% on average.

## 5. Discussion

### 5.1. Limitations of Sensing

For all three types of micromotion considered in this study, the proposed method accurately detected their frequency and duration of motion. However, the comparison with the reference indicated some limitations and errors.

Theoretically, the magnitude of the vibration of the steel ruler decreases gradually from the initial 10 cm deflection at the free end. However, the variation in the CSI amplitude in response to the vibration of the steel ruler did not represent the decay trend. By analyzing the waveform changes in CSI, we detected the frequency and duration of the vibration accurately, demonstrating the ability to quantify the motion on a centimeter scale. However, it is challenging to determine the amplitude of the vibration. Wireless signals are subject to complex multipath effects during their propagation. Even with the same type of movement and amplitude, the signals cannot always reach the Rx with the same intensity.

In the finger-tapping tests, the number of taps was accurately counted when the direction of motion was perpendicular to the LoS. However, when the direction of motion was parallel to the LoS, we could not obtain the number of taps, but could roughly distinguish between the active and non-active states. This is because, according to the Fresnel zone theory, only movements along the direction normal to the Fresnel zone affect the reflection path, whereas those in the tangential direction would have no influence [57]. Interestingly, in the steel ruler vibration test, we detected the number of vibrations in both the perpendicular and parallel cases. This is likely because the ruler’s size and motion range were relatively larger than those of the fingers. The entire ruler (40 cm) was in action during the vibration and had more non-tangential movements than finger tapping. In contrast, finger tapping only involved two fingers (shorter than 10 cm) in action with a centimeter-level motion, which is small. Moreover, the steel material is more reflective of the WiFi signal and has a larger reflective area than the fingers. Thus, the vibration is detectable despite a small motion.

**Figure 15:**
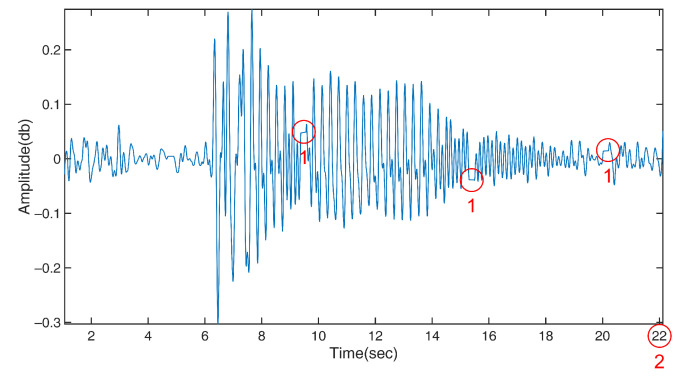
Illustration of packet loss (red Type 1 circles) and overestimation of the motion duration (red Type 2 circle).

Errors were also caused by packet loss, the slow response of the wireless signal, and the multipath effect. Packet loss commonly occurs during the transmission of wireless signals, and is unavoidable. When packet loss occurred in our tests, the Rx still recorded CSI at the specified rate (i.e., 1000 Hz) and only logged the same value as the previous data point, leading to a horizontal line shown in the signal plot (Figure 15). In the figure, the three spots marked by red Type 1 circles denote the situation of packet loss, which could confuse the computer algorithm used for identifying peaks. The packet loss also caused an overestimation of the motion duration (red Type 2 circle). The significance of errors depends on the severity of the packet loss. However, packet loss is easy to detect due to its feature of logging the same data value as the previous one. The algorithm should detect packet loss and avoid using the data during its occurrence. Moreover, the communication rate of the WiFi pair and CSI sampling rate can be optimized for the devices to find the best match at the required data resolution.

**Figure 16:**
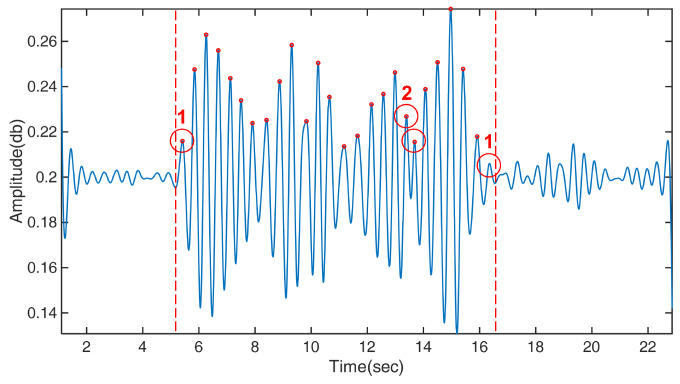
Illustration of two types of errors: slow response (Type 1 circles) and two peaks in the same half wave (Type 2 circles). The smaller red circles indicate peaks in the data identified by our algorithm and are used to count the number of movements.

A slow response indicates that the wireless signal does not immediately respond to the impact of the motion at its start and end. As shown in Figure 16, two smaller waves were produced at the start and end of the active state, as indicated by two red Type 1 circles. Each of these smaller waves also represents a movement, which may not be recognized by our algorithm, leading to the miscalculation of the number of movements. In addition, the multipath effect resulted in two peaks in the same half wave, as shown by the red Type 2 circles. In this example, both peaks were miscounted as movements.

The input CSI sequence was controlled in the same length (20 s) for all scenarios used to evaluate the proposed approach. Since the input CSI sequence length was short and fixed, the data processing algorithm read it all at once to segment the target motion signal without concerning the computation performance. However, in a real situation, target motion can occur at any point in the received CSI data streams, which requires a method to identify data frames in the CSI stream that contain motion signals. Future works should develop a motion recognition method to identify target motion occurring at a random time point and with an arbitrary time length. The block-processing method would be more feasible and efficient for such an implementation than the sample-processing method. For real-time applications, the delay due to block processing should be considered.

### 5.2. Performance of Sensing and Applicability

Overall, the accuracy of estimating the motion duration was much higher than that of determining the motion frequency, and the standard deviation of the former was lower than that of the latter. This can be explained by the error sources. As shown in Figure 16, both types of errors affect the peak identification, causing an incorrect calculation of the motion frequency. When these errors occur, more fake peaks are incorrectly calculated to cause an overestimation. Moreover, only the Type 1 error produced a discrepancy in the estimation of the motion duration. This is because the determination of motion duration only requires distinguishing between the active and inactive states in the signal. While the Type 2 error created additional peaks in the middle of the data, it did not extend or compress the total time of the active state. Therefore, there were fewer types of error sources to create artifacts in the motion duration estimation, leading to a better sensing performance.

The frequency spectrum of the CSI data could contain useful information to help improve the sensing performance. A fast Fourier transform of the resting tremor motion (Figure 8g) indicated that major component frequencies were between 2 and 5 Hz, close to the range of motion frequency (3–6 Hz) instructed to perform in the tests. Movement disorders like tremors and bradykinesia have irregular patterns that differ between individuals. The frequency domain analysis can provide additional information to help identify the target motion and improve noise filtering to enhance the data quality.

A past study realized a system that can detect subtle motion, i.e., human respiration, in a range of distances [44]. Instead of using basic signals of CSI, the system employed the CSI ratio, the ratio of CSI readings from two antennas, to cancel out the noise and enhance the sensing performance. We conducted the resting tremor test with six repetitions (Figure 4c) to compare the sensing performance when using the CSI amplitude and CSI ratio of amplitudes processed by our workflow. As shown in Table 5, either type of CSI data could be used to achieve high accuracy in the sensing duration and frequency of motion, but the CSI ratio needed data received from a WiFi device equipped with two or more antennas. However, it should be noted that the comparison was not intended to evaluate the performance of system [44] as its main purpose was to extend the range limit of motion sensing.

This study showed that different types of subtle hand motion, i.e., finger tapping and resting tremor, could be accurately quantified, and their features of motion could be captured in the CSI signal variation. After the data processing steps, the known range of the target motion frequency could be used to calculate the minimum separation, which was combined with the empirically determined minimum peak height and prominence to accurately quantify the motion. The literature may have provided sufficient information to make this scenario applicable to on-field detection for motor evaluation. Features of different types of tremors (frequency, amplitude, pattern, and distribution) have been studied [58]. Features of finger tapping, such as speed and tapping intervals, were found to correlate well with standard rating scales such as MDS-UPDRS [59]. Therefore, these known features and correlations can be used to establish patient-specific criteria for more accurate motion quantification. However, the aforementioned scenario would require pre-configuration when the proposed method is introduced to a new environment or subject. For generalization purposes, it is critical to analytically define the features of motion, making data collection with a variety of subjects and environments an important future work.

The sensing accuracy decreased linearly when the motion moved away from the LoS in the Y-direction. In the X-direction, the accuracy was much less sensitive to the change in the distance to the LoS. Wireless signals fade during propagation owing to environmental effects, phase interference, and phase time variability between signals. When the resting tremor occurred farther away from the LoS, the received data were more disturbed by the signal reflection. If environmental noise gradually dominates the signal presentation, the effect of tremor motion becomes trivial, increasing the quantification error.

When three Rxs were paired with a Tx and arranged at the four corners of the room, three Fresnel zones were formed. All experimental points were covered by at least one Fresnel zone with good signal reception; thus, the average sensing accuracy was higher than when using a single Rx. However, single-Rx detection reached a higher maximum accuracy than multi-Rx detection. This can be attributed to the application of PCA in the data processing. For multi-Rx detection, the data of all 180 subcarriers from three Rxs were processed together through PCA, resulting in an averaged performance of the final signal. In the future, a subcarrier selection process should be added before PCA to eliminate unnecessary data interfering with the performance of PCA. Furthermore, the errors found at the six validation points were all within 2.5%, confirming the symmetric property of the sensing accuracy contour.

This study investigated two common Tx-Rx arrangements, and the sensing performance could be understood from the accuracy contours (Figure 10, Figure 11, Figure 12, Figure 13 and Figure 14). Based on the Fresnel zone theory, the effect of WiFi link positioning and room size can be predicted. When there was a single WiFi link in the room, a single elliptical Fresnel zone with Tx and Rx located at the two focal points was formed [57]. Moving Tx and Rx in the direction parallel to the LoS mainly changes the distance of the foci and ellipse profile, which is expected to make a relatively small difference in the accuracy contour. When moving Tx and Rx in the direction perpendicular to the LoS, e.g., to the middle of the room, the accuracy contour is expected to be symmetric to not only the centerline of LoS but also the LoS. When changing to a wider or longer room, the sensing accuracy would reduce further at locations closer to the four edges. A nonlinear reduction is expected as the Fresnel zone is elliptical. When extending to multiple WiFi links, the contour of sensing accuracy is a combined effect of their Fresnel zones, in which originally weak sensing spots of the single WiFi link can be enhanced by the second or third link. Therefore, the reduction effect should be less significant when using multiple WiFi links.

Signal disturbances are commonly found in healthcare environments. A comprehensive evaluation is needed in the future to move the proposed approach closer to a real-life application. A preliminary study was conducted to investigate the impact of static and dynamic objects on the sensing performance. We placed an office partition wall constructed from an aluminum frame and wood as a static barrier to block the LoS. Resting tremor tests were performed with and without the barrier. To simulate dynamic disturbance, a person was asked to walk back and forth near (approximately the same distance as the target motion from the LoS but on the opposite side of LoS) and far (3 m) away from the LoS. Another person performed resting tremor tests with and without dynamic disturbance. The results showed that the sensing accuracy remained the same for the duration of motion but reduced from 91.7% to 72.3% for the frequency of motion under the presence of the static barrier. When the dynamic disturbance was introduced, human walking near the LoS dominated the signal response, making the target motion not quantifiable; walking on the far side of the LoS had a significantly reduced effect, so the sensing accuracy was around the same for the duration of motion and reduced by 3.1% for the estimation of motion frequency compared to the case without disturbance. In summary, the biggest challenge to overcome is when a large motion occurs nearby the LoS to shield the target motion, which is currently an open issue. For health monitoring applications, an alternative solution might be to first recognize target motion from the CSI data and discard the unrecognizable data segments. With an accurate recognition algorithm, the monitoring system should still be able to collect a significant amount of reliable data for quantification. As an assessment tool, clear instructions can be given to set up the environment to properly avoid disturbance.

## 6. Conclusions

This paper demonstrates a WiFi-based approach that enables the contactless detection of micromotion related to hand movement disorders of PD. This approach accurately quantifies the frequency and duration of the hand resting tremor and finger tapping, which are critical indicators of the disease. Moreover, we confirmed that the micromotion could be detected in a wide space by showing experimentally verified contours of sensing accuracy for the arrangements of single and multiple links.

The proposed approach can be implemented as a clinical assessment tool to detect the patient’s movements simultaneously when the doctor conducts a regular motor evaluation. The doctor can review and compare the quantified movements with their evaluation. The proposed approach could also be further developed for telemedicine applications to monitor a patient’s movements in daily life and track drug efficacy by providing quantitative data on symptoms. Other than clinical applications, gesture recognition could be benefited by analyzing the subtle features in the quantified hand and finger movements; after some parameter tuning, other types of subtle motion, such as eating or breathing, could be detected. WiFi-based motion detection is a device-free and passive sensing method that allows for capturing long-term data under the patient’s natural condition to provide doctors with a comprehensive insight into the disease status, leading to a more timely and personalized treatment that will improve the quality of life and slow the disease progression in a patient.

## Figures and Tables

**Table 1:** Parameters used in the CSI data processing procedures for quantifying micromotion.

Data Processing Procedure	Parameter
Butterworth filter	**Finger tapping:** *n* = 3 $f_{l}$ = 1 Hz, $f_{h}$ = 10 Hz b = [2.1378, 0, −6.4133, 0, 6.4133, 0, −2.1378] $\times \;10^{-5}$ a = [1, −5.8858, 14.4363, −18.8875, 13.9021, −5.4582, 0.8931] **Rest tremor:** *n* = 3 $f_{l}$ = 3 Hz, $f_{h}$ = 6 Hz b = [0.8216, 0, −2.4648, 0, 2.4659, 0, −0.8216] $\times \;10^{-6}$ a = [1, −5.9602, 14.8038, −19.6133, 14.6189, −5.8123, 0.9630]
Short-time energy threshold segmentation	N = 51
Savitzky–Golay smoothing filter	*n* = 3 *m* = 101

**Table 2:** Descriptions of the three types of motion and features expected to see in their signals.

Target Motion	Characteristics of Motion
Steel Ruler Vibration	The ruler with a sensor (MetaMotionC, MBIENTLAB Inc., San Francisco, CA, USA) attached to one end oscillates with a 10 cm initial displacement and decreases its amplitude over time until it returns to the static condition. The signal of motion is a sine wave that gradually decays until the motion becomes too small to be detectable. The frequency of oscillation measured by the sensor was 3.46 Hz.
Finger Tapping	A person repeatedly taps the tip of the index finger against the tip of the thumb at approximately 1 Hz. The signal of motion shows a strong impulse at each tap, but lower responses during the movements of fingers.
Hand Resting Tremor	A person moves the palm back and forth about the axis of the arm by rotating the wrist with random frequencies in the range of 3–6 Hz. The signal of motion is similar to oscillations with irregular intervals. The signal strength should be higher than finger tapping as the motion is larger.

**Table 3:** Detailed experimental settings and types of target motion used in each scenario.

	a	b	c	d
Tx-Rx arrangement	Single link	Single link	Single link	Multiple links
Tx/Rx (no. of devices, no. of antennas for each device)	(1,2)/(1,3)	(1,2)/(1,3)	(1,2)/(1,3)	(1,2)/(3,1)
Carrier frequency	5 GHz	5 GHz	5 GHz	5 GHz
Bandwidth	20 MHz	20 MHz	20 MHz	20 MHz
Sampling rate	1000 Hz	1000 Hz	1000 Hz	1000 Hz
Number of CSI streams	6	6	6	6
Number of CSI subcarrier per stream	30	30	30	30
Transmit/Receive mode	Receive mode	Receive mode	Receive mode	Receive mode
Target motion	RV, FT, RT Figure 4a–c	RV, FT Figure 4a,b,d,e	RT Figure 4c	RT Figure 4c
Distance between target motion and LoS	1 m	1 m	X: 0–4 m, Y: 0–6 m	X: 0–4 m, Y: 0–6 m
Length for each data sequence of motion	20 s	20 s	20 s	20 s
Number of data samples in each sequence of motion	1000 × 20	1000 × 20	1000 × 20	1000 × 20
Number of data sequences collected for each type of target motion	8	8	8	8

RV: steel ruler vibration, FT: finger tapping, RT: hand-resting tremor.

**Table 4:** Sensing accuracy of the proposed approach for detecting the three types of motion perpendicular and parallel to LoS.

Motion Type	Steel Ruler Vibration		Finger Tapping		Resting Tremor	
**Direction to LoS**	**Perp.**	**Hori.**	**Perp.**	**Hori.**		
Duration	-	-	98.7%	97.6%	97.3%	
Frequency	96.2%	86.4%	98.7%	-	91.7%	

**Table 5:** A comparison of sensing accuracy when detecting hand resting tremor using CSI amplitude and CSI ratio.

	CSI Amplitude	CSI Ratio
Duration	98.7 ± 1.3%	96.5 ± 0.8%
Frequency	94.2 ± 3.3%	97.6 ± 2.1%

## Data Availability

Not applicable.

## References

[1] Qian K., Wu C., Zhang Y., Zhang G., Yang Z., Liu Y. Widar2.0: Passive Human Tracking with a Single Wi-Fi Link. Proceedings of the 16th Annual International Conference on Mobile Systems, Applications, and Services (MobiSys ’18).

[2] Wu D., Zhang D., Xu C., Wang H., Li X. (2017). Device-free WiFi human sensing: From pattern-based to model-based approaches. IEEE Commun. Mag..

[3] Zeng Y., Pathak P.H., Mohapatra P. WiWho: WiFi-Based Person Identification in Smart Spaces. Proceedings of the 15th ACM/IEEE International Conference on Information Processing in Sensor Networks (IPSN).

[4] Al-qaness M.A.A., Li F., Ma X., Zhang Y., Liu G. (2016). Device-Free Indoor Activity Recognition System. Appl. Sci..

[5] Wang Y., Wu K., Ni L.M. (2017). WiFall: Device-Free Fall Detection by Wireless Networks. IEEE Trans. Mob. Comput..

[6] Grandez K., Bustamante P., Solas G., Gurutzeaga I., García-Alonso A. Wearable wireless sensor for the gait monitorization of Parkinsonian patients. Proceedings of the 16th IEEE International Conference on Electronics, Circuits and Systems.

[7] Niazmand K., Tonn K., Kalaras A., Kammermeier S., Boetzel K., Mehrkens J.H., Lueth T.C. A measurement device for motion analysis of patients with Parkinson’s disease using sensor based smart clothes. Proceedings of the 5th International Conference on Pervasive Computing Technologies for Healthcare (PervasiveHealth) and Workshops.

[8] Holmes J., Jenkins M., Johnson A., Hunt M., Clark R. (2013). Validity of the Nintendo Wii® balance board for the assessment of standing balance in Parkinson’s disease. Clin. Rehabil..

[9] Bhat G., Deb R., Ogras U.Y. (2019). OpenHealth: Open-Source Platform for Wearable Health Monitoring. IEEE Des. Test.

[10] Dranca L., Mendarozketa L., Goñi A., Illarramendi A., Gomez I., Alvarado M., Rodríguez-Oroz M. (2018). Using Kinect to classify Parkinson’s disease stages related to severity of gait impairment. BMC Bioinform..

[11] Zhu W., Anderson B., Zhu S., Wang Y. A Computer Vision-Based System for Stride Length Estimation using a Mobile Phone Camera. Proceedings of the 18th International ACM SIGACCESS Conference on Computers and Accessibility.

[12] Zhang Z., Ishida S., Tagashira S., Fukuda A. (2019). Danger-Pose Detection System Using Commodity WiFi for Bathroom Monitoring. Sensors.

[13] Ma Y., Zhou G., Wang S. (2019). WiFi sensing with channel state information: A survey. ACM Comput. Surv..

[14] Yousefi S., Narui H., Dayal S., Ermon S., Valaee S. (2017). A Survey on Behavior Recognition Using WiFi Channel State Information. IEEE Commun. Mag..

[15] Wang Z., Hou Y., Dou W., Zhang C., Huang Z., Guo Y. (2019). A Survey on Human Behavior Recognition Using Channel State Information. IEEE Access.

[16] Wang Z., Huang Z., Zhang C., Dou W., Guo Y., Chen D. (2021). CSI-based human sensing using model-based approaches: A survey. J. Comput. Des. Eng..

[17] Moustafa A.A., Chakravarthy S., Phillips J.R., Gupta A., Keri S., Polner B., Frank M.J., Jahanshahi M. (2016). Motor symptoms in Parkinson’s disease: A unified framework. Neurosci. Biobehav. Rev..

[18] Wu D., Zhang D., Xu C., Wang Y., Wang H. WiDir: Walking direction estimation using wireless signals. Proceedings of the 2016 ACM International Joint Conference on Pervasive and Ubiquitous Computing (UbiComp ’16).

[19] Bächlin M., Plotnik M., Roggen D., Maidan I., Hausdorff J.M., Giladi N., Tröster G. (2009). Wearable Assistant for Parkinson’s Disease Patients With the Freezing of Gait Symptom. IEEE Trans. Inf. Technol. Biomed..

[20] Salarian A., Russmann H., Wider C., Burkhard P.R., Vingerhoets F.J., Aminian K. (2007). Quantification of tremor and bradykinesia in Parkinson’s disease using a novel ambulatory monitoring system. IEEE Trans. Biomed. Eng..

[21] LeMoyne R., Mastroianni T., Cozza M., Coroian C., Grundfest W. Implementation of an iPhone for characterizing Parkinson’s disease tremor through a wireless accelerometer application. Proceedings of the 2010 Annual International Conference of the IEEE Engineering in Medicine and Biology Society.

[22] López-Blanco R., Velasco M.A., Méndez-Guerrero A., Romero J.P., Castillo M.D.D., Serrano J.I., Rocon E., Benito-León J. (2019). Smartwatch for the analysis of rest tremor in patients with Parkinson’s disease. J. Neurol. Sci..

[23] Khatsenko K., Khin Y., Maibach H. (2020). Allergic Contact Dermatitis to Components of Wearable Adhesive Health Devices. Dermatitis.

[24] Kour N., Sunanda, Arora S. (2019). Computer-Vision Based Diagnosis of Parkinson’s Disease via Gait: A Survey. IEEE Access.

[25] Pang Y., Christenson J., Jiang F., Lei T., Rhoades R., Kern D., Thompson J.A., Liu C. (2020). Automatic detection and quantification of hand movements toward development of an objective assessment of tremor and bradykinesia in Parkinson’s disease. J. Neurosci. Methods.

[26] Cho C.W., Chao W.H., Lin S.H., Chen Y.Y. (2009). A vision-based analysis system for gait recognition in patients with Parkinson’s disease. Expert Syst. Appl..

[27] Krupicka R., Szabo Z., Viteckova S., Ruzicka E. (2014). Motion capture system for finger movement measurement in Parkinson disease. Radioengineering.

[28] Agostino R., Currà A., Giovannelli M., Modugno N., Manfredi M., Berardelli A. (2003). Impairment of individual finger movements in Parkinson’s disease. Mov. Disord..

[29] Chang C., Huang Y., Chen J., Lee C. Improving Automatic Tremor and Movement Motor Disorder Severity Assessment for Parkinson’s Disease with Deep Joint Training. Proceedings of the 41st Annual International Conference of the IEEE Engineering in Medicine and Biology Society.

[30] Ghosh A., Sanyal A., Chakraborty A., Sharma P.K., Saha M., Nandi S., Saha S. On automatizing recognition of multiple human activities using ultrasonic sensor grid. Proceedings of the 9th International Conference on Communication Systems and Networks.

[31] Liakat S., Bors K., Xu L., Woods C., Doyle J., Gmachl C. (2014). Noninvasive in vivo glucose sensing on human subjects using mid-infrared light. Biomed. Opt. Express.

[32] Fan L., Li T., Yuan Y., Katabi D. (2020). In-Home Daily-Life Captioning Using Radio Signals. arXiv.

[33] Shi S., Sigg S., Ji Y. Joint localization and activity recognition from ambient FM broadcast signals. Proceedings of the 2013 ACM International Joint Conference on Pervasive and Ubiquitous Computing.

[34] Zhong Y., Zhou Z., Jiang T. The Recognition of Human Activities Under UWB Communication. Proceedings of the Third International Conference on Communications, Signal Processing, and Systems.

[35] Depatla S., Mostofi Y. Crowd Counting through Walls Using WiFi. Proceedings of the 2018 IEEE International Conference on Pervasive Computing and Communications.

[36] Wang Z., Guo B., Yu Z., Zhou X. (2018). Wi-Fi CSI-Based Behavior Recognition: From Signals and Actions to Activities. IEEE Commun. Mag..

[37] Halperin D., Hu W., Sheth A., Wetherall D. (2011). Tool release: Gathering 802.11 n traces with channel state information. ACM SIGCOMM Comput. Commun. Rev..

[38] Wang Y., Liu J., Chen Y., Gruteser M., Yang J., Liu H. E-eyes: Device-free location-oriented activity identification using fine-grained WiFi signatures. Proceedings of the 20th Annual International Conference on Mobile Computing and Networking (MobiCom’14).

[39] Youssef M., Mah M., Agrawala A. Challenges: Device-free passive localization for wireless environments. Proceedings of the 13th Annual ACM International Conference on Mobile Computing and Networking (MobiCom ’07).

[40] Zhang J., Wei B., Hu W., Kanhere S.S. WiFi-ID: Human Identification Using WiFi Signal. Proceedings of the 2016 International Conference on Distributed Computing in Sensor Systems (DCOSS).

[41] Li H., Yang W., Wang J., Xu Y., Huang L. WiFinger: Talk to your smart devices with finger-grained gesture. Proceedings of the 2016 ACM International Joint Conference on Pervasive and Ubiquitous Computing (UbiComp ’16).

[42] Wang G., Zou Y., Zhou Z., Wu K., Ni L.M. (2016). We Can Hear You with Wi-Fi!. IEEE Trans. Mob. Comput..

[43] Lin Z., Xie Y., Guo X., Ren Y., Chen Y., Wang C. WiEat: Fine-grained Device-free Eating Monitoring Leveraging Wi-Fi Signals. Proceedings of the 29th International Conference on Computer Communications and Networks (ICCCN).

[44] Zeng Y., Wu D., Xiong J., Yi E., Gao R., Zhang D. FarSense: Pushing the Range Limit of WiFi-based Respiration Sensing with CSI Ratio of Two Antennas. Proceedings of the ACM on Interactive, Mobile, Wearable and Ubiquitous Technologies.

[45] Mo H., Kim S. (2021). A Deep Learning-Based Human Identification System with Wi-Fi CSI Data Augmentation. IEEE Access.

[46] Zhang F., Niu K., Xiong J., Jin B., Gu T., Jiang Y., Zhang D. (2019). Towards a diffraction-based sensing approach on human activity recognition. ACM Interact. Mob. Wearable Ubiquitous Technol..

[47] Soltanaghaei E., Kalyanaraman A., Whitehouse K. Peripheral WiFi vision: Exploiting multipath reflections for more sensitive human sensing. Proceedings of the 4th International on Workshop on Physical Analytics.

[48] Wang W., Liu A.X., Shahzad M., Ling K., Lu S. Understanding and Modeling of WiFi Signal Based Human Activity Recognition. Proceedings of the 21st Annual International Conference on Mobile Computing and Networking (MobiCom ’15).

[49] Yang X., Shah S.A., Ren A., Zhao N., Zhang Z., Fan D., Zhao J., Wang W., Ur-Rehman M. (2018). Freezing of gait detection considering leaky wave cable. IEEE Trans. Antennas Propag..

[50] Shah S.A., Yang X., Abbasi Q.H. (2019). Cognitive health care system and its application in pill-rolling assessment. Int. J. Numer. Model. Electron. Netw. Devices Fields.

[51] Butterworth S. (1930). On the Theory of Filter Amplifiers. Exp. Wirel. Wirel. Eng..

[52] BMJ Evaluation of Tremor. https://bestpractice.bmj.com/topics/en-us/974.

[53] Bachu R.G., Kopparthi S., Adapa B., Barkana B.D. (2010). Voiced/Unvoiced Decision for Speech Signals Based on Zero-Crossing Rate and Energy. Adv. Tech. Comput. Sci. Softw. Eng..

[54] Savitzky A., Golay M.J. (1964). Smoothing and Differentiation of Data by Simplified Least Squares Procedures. Anal. Chem..

[55] Sen S., Radunović B., Choudhury R.R., Minka T. You are facing the Mona Lisa:spot localization using PHY layer information. Proceedings of the 10th International Conference on Mobile Systems, Applications, and Services.

[56] Neter J., Kutner M.H., Nachtsheim C.J., Wasserman W. (1996). Applied Linear Statistical Models.

[57] Wang H., Zhang D., Ma J., Wang Y., Wang Y., Wu D., Gu T., Xie B. Human respiration detection with commodity wifi devices: Do user location and body orientation matter?. Proceedings of the 2016 ACM International Joint Conference on Pervasive and Ubiquitous Computing.

[58] Surangsrirat D., Sri-iesaranusorn P., Chaiyaroj A., Vateekul P., Bhidayasiri R. (2022). Parkinson’s disease severity clustering based on tapping activity on mobile device. Sci. Rep..

[59] Goetz C., Tilley B., Shaftman S., Stebbins G., Fahn S., Martinez-Martin P., Poewe W., Sampaio C., Stern M., Dodel R. (2019). MDS-Unified Parkinson’s Disease Rating Scale (MDS-UPDRS).

